# Supplementary material for: ReMASTER: improved phylodynamic simulation for BEAST 2.7
Source: Bioinformatics. 2024 Jan 9;40(1):btae015. doi: 10.1093/bioinformatics/btae015 (PMC10796175; doi:10.1093/bioinformatics/btae015)
Supplement: btae015_Supplementary_Data [file btae015_supplementary_data.pdf]

Supplemental Text for "*ReMASTER:* Improved phylodynamic simulation for BEAST 2.7"

Timothy Vaughan

1 Running time comparison study details

To compare the running times of ReMASTER's birth-death tree simulation with those of MASTER, I defined a simple single-type stochastic-logistic model with the following reactions:

$$X \xrightarrow{\beta} 2X$$
$$2X \xrightarrow{\gamma} X$$

where β and γ are the birth and death rates, respectively. Since it lacks an absorbing state, this model has a well-defined stationary distribution around the carrying capacity $N_c = 2\beta/\gamma$.

To perform the comparison, both MASTER and ReMASTER simulated reconstructed 100-tip trees under this model. These leaves were sampled at a fixed number of time units following the start of the simulation. In MASTER the leaves were produced using the `LineageSampler` class, as described in the the MASTER documentation. In ReMASTER, leaves were produced using a punctual reaction at the appropriate time:

$$X \xrightarrow{N=100} sample.$$

Simulated trees were produced using both packages using a fixed decay rate of 0.01 and a variety of birth rates, starting at 1 and increasing roughly exponentially. The total simulated time duration was fixed at 100 time units to ensure that the population remained in its equilibrium state for the majority of the simulation. The time taken for each simulation was then recorded.

Rather than compare these run times to the associated birth rates or carrying capacities—which are overly specific to this particular model and do not

adequately reflect the size of the computational task—it is more useful to consider how they depend on the number of trajectory events that are being calculated in each simulation. This can be roughly estimated by considering that the total rate $\rho(\beta, \gamma)$ at which reactions occur in the equilibrium situation is simply the sum of the individual birth and death reaction propensities for a population of that size, i.e.

$$\begin{aligned}\rho(\beta,\gamma) &\simeq \beta N_c + \gamma N_c^2/2\\ &= \frac{4\beta^2}{\gamma}\end{aligned}$$

By multiplying this overall rate by the total simulated time of 100 time units, we can further estimate the total number of events produced by a single equilibrium stochastic logistic model trajectory to be approximately $100\rho(\beta, 0.01) = 4 \times 10^4 \times \beta^2$.

Figure 1 in the manuscript shows the run times required by the two packages against the corresponding estimate of the number of trajectory events simulated for each birth rate value.

The simulation and plotting scripts used to produces these results are available from the author's GitHub repository at `https://github.com/tgvaughan/remaster-master-comparison`.
